# Reset Tree-Based Optical Fault Detection

**DOI:** 10.3390/s130506713

**Published:** 2013-05-21

**Authors:** Dong-Geon Lee, Dooho Choi, Jungtaek Seo, Howon Kim

**Affiliations:** 1 Computer Engineering Department, Pusan National University, Busan 609-735, Korea; E-Mail: guneez@pusan.ac.kr; 2 Electronic and Telecommunications Research Institute (ETRI), Daejeon 305-700, Korea; E-Mail: dhchoi@etri.re.kr; 3 The Attached Institute of ETRI, Daejeon 305-811, Korea; E-Mail: seojt@ensec.re.kr

**Keywords:** optical fault, single event transient, soft-error, reset tree, fault detection

## Abstract

In this paper, we present a new reset tree-based scheme to protect cryptographic hardware against optical fault injection attacks. As one of the most powerful invasive attacks on cryptographic hardware, optical fault attacks cause semiconductors to misbehave by injecting high-energy light into a decapped integrated circuit. The contaminated result from the affected chip is then used to reveal secret information, such as a key, from the cryptographic hardware. Since the advent of such attacks, various countermeasures have been proposed. Although most of these countermeasures are strong, there is still the possibility of attack. In this paper, we present a novel optical fault detection scheme that utilizes the buffers on a circuit's reset signal tree as a fault detection sensor. To evaluate our proposal, we model radiation-induced currents into circuit components and perform a SPICE simulation. The proposed scheme is expected to be used as a supplemental security tool.

## 1. Introduction

Fault injection is one of the most threatening types of attack, revealing information in a cryptographic integrated circuit (IC) by causing chip malfunction. Secrets are then disclosed using faulty results from the flipped bits in the circuit logic or manipulated conditional jumps to reduce the encryption rounds.Usually, voltage and clock glitches [1–4] are used as a fault source on the chip. Strong light beams, such as lasers or flashlights, are also used to cause abnormal behavior in the silicon device [5–8]. Many attacks have been introduced to target the famous RSA [9] and AES [10].

Among the attacks listed above, optical fault injection removes the package that protects the IC before firing a light beam, such as laser, ultra violet(UV), or infrared(IR) light, which causes a malfunction. When a high-energy light beam strikes a silicon device, it generates electron-hole pairs, which give rise to transient currents [11]. This may cause no notable effect, a transient bit-flip or state change, or permanent damage to the device. Temporal state changes, known as single-event transients or soft-errors, have been studied for a long time in the fields of nuclear science and integrated circuits. Cosmic ray-induced upsets are observed in ICs used in aerospace, such as artificial satellites. To increase chip reliability, methods to simulate these phenomena [12,13] and countermeasures to protect the devices [14–16] have been studied. The importance of this research increased after these effects were observed not only in aerospace but also at sea level. As they have started to be used in attacks against cryptographic hardware, these effects are now studied in the field of cryptography.

To date, many fault injection attacks and their subsequent countermeasures have been introduced. However, most of these countermeasures are not complete and are still vulnerable to attack. In this paper, we present a novel reset tree-based optical fault detection (RTOFD) technique to strengthen cryptographic hardware against such attacks. Since our scheme utilizes the circuit's buffers over the reset tree as an optical sensor, it provides higher security with low logical cost.

The rest of this paper is organized as follows. In Section 2, various optical fault attacks and their countermeasures are introduced. The proposed RTOFD scheme is explained in Section 3, and its effectiveness is discussed in Section 4. Effects and limitations of the proposed scheme are discussed in Section 5 and finally, we conclude this paper in Section 6.

## 2. Optical Fault Injection Attacks

In this section, we introduce various optical fault injection techniques that target cryptographic hardware. We then consider the various light sources used to induce faults.

### 2.1. Attacks Against Cryptographic Hardware

Generally, the security of a cryptographic algorithm is determined using mathematical methods. However, attacks that exploit unintended vulnerabilities caused by hardware implementation have recently been reported. One famous type of attack is side-channel analysis, which collects side-channel information leaked from cryptographic hardware and reveals secret information held in the device. Probe attacks [17], which read signals in the IC by attaching a very thin probe to the metal line, have also been documented. The layout of the circuit can be used to analyze the logic structure. Another threatening type of attack is fault injection, which reveals secret information in a cryptographic IC by causing a malfunction in the chip. We will deal with this fault injection attack in our paper.

In this subsection, we first introduce the RSA [9] and RSA–CRT [18] algorithms, before presenting Bellcore's fault injection attack scheme against RSA–CRT.

#### 2.1.1. RSA Algorithm

The parameters and keys used in the RSA algorithm are calculated as follows:

1. Choose random prime numbers *p* and *q*.
2. Calculate *n* = *pq*.
3. Choose *e* as a public key, where *e* < *ϕ*(*n*), *GCD*(*ϕ*(*n*), *e*) = 1, and *ϕ* is Euler's totient function.
4. Use *d* as a private key where *e* · *d* ≡ 1 mod *ϕ*(*n*).

Of these parameters, *e* is public and *p*, *q*, and *d* should be kept secret. The encryption (verification of signature) process is expressed as *c* = *m^e^* mod *n*, and the decryption (signing) process is given by *m* = *c^d^* mod *n*, where *m* is plaintext and *c* is cipher text.

#### 2.1.2. RSA-CRT Algorithm

The most time-consuming part of the RSA calculation is exponentiation. As *n* grows, the calculation time for exponentiation gets longer. To shorten the calculation time, RSA-CRT [18] was introduced. By splitting *n* into *p* and *q* and using CRT (Chinese Remainder Theorem), the cipher calculation can be sped up approximately 4 times. The RSA-CRT scheme can be used when the private key is known (during the signing and decryption). The CRT recombination function *z* = *CRT*(*x, y*) mod *n* is defined as *z* ≡ *x* mod *p*, *z* ≡ *x* mod *q*, where *z* ∈ *Z*/*nZ*. The sign *s* can be calculated as follows:

1. *s_p_* = *m*^*d mod* (*p*-1)^ mod *p*.
2. *s_q_* = *m*^*d mod* (*q*-1)^ mod *q*.
3. *s* = *CRT* (*s_p_*, *s_q_*) = ((*s_q_*- *s_p_*) · *p*^-1^ mod *q*)· *p* + *s_p_*.

#### 2.1.3. Fault Attacks on RSA-CRT

The first fault attack on RSA-CRT was introduced in [19]. If a fault is injected during the calculation of *s_p_*, the result will be contaminated. We denote this by $s_{p}^{\prime }$. The *s′* is calculated as

(1) $$s^{\prime }=((s_{q}-s_{p}^{\prime }).p^{-1}\mathrm{mod}q).p+s_{p}^{\prime }$$

If we calculate gdc(*s* − *s*′, *n*), then the secret value *q* can easily be revealed because *s* = *s′* mod *q* and *s* ≠ *s′* mod *p*.

### 2.2. Optical Fault Injection Methods

In this subsection, we introduce the various types of light beam used for attacks, and present their characteristics and attack methods.

#### 2.2.1. Flashlight

Skorobogatov and Anderson [5,6] used a camera flashlight as a very cheap light source in order to change the contents of SRAM. In their experiment, they bought a *£*20 used flashlight at a second-hand market. They decapped Microchip's PIC16F84 microcontroller and attacked the SRAM in the device. Aluminum foil was used as an aperture in a successful attack on each cell in the SRAM.

#### 2.2.2. Laser

Laser is one of the most widely used light sources of injecting faults into a chip. A laser is a narrow, concentrated high-energy light beam that is spatially coherent. These characteristics make it useful for fault-injection against cryptographic devices. Originally, lasers were used to generate radiation-induced current-like effects on semiconductors [20]. Following that, an optical-beam-induced current method was introduced. This method scans the semiconductor die to detect any defects on the chip [21], using the current from the electron-hole pair caused by an electron strike.

Skorobogatov and Anderson also introduced the first laser attack [5,6]. They used the same device from their flash attack, and shot a laser into the SRAM region. In their laser injection experiments, they succeeded in flipping each bit using only a *£*5 laser pointer with 5 mW of power and a 650 nm wavelength. The spot of the laser was 1 μm^2^, which was enough for cell-based access.

In 2010, Trichina and Korkikyan [7] attacked a fault attack-resilient RSA–CRT implementation. The RSA–CRT algorithm was incorporated into an ARM Cortex M3 core with countermeasures against a fault injection attack. The authors twice injected a laser and successfully disarmed the countermeasures. In their experiment, they used a relatively inexpensive YAG laser with a spot size of 1–2 μm^2^ and a wavelength of 1,064 nm. They mounted the target chip on a driving board and moved it along the X and Y axes by 1 μm.

Wonderberg *et al.* formulated an attack using a diode laser with a good trigger signal response [8]. The laser operated at the 808 nm and 1,064 nm wavelengths. Lenses were used to concentrate the light beam into a 6 μm ×1.4 μm spot. In contrast to general attack methods, which inject light through the top of the die, they shot the laser through the silicon substrate on the bottom of the chip. This is because the top of the die is usually covered with a metal shield. To penetrate the substrate, they used the 1,064 nm laser. The power consumption pattern was analyzed along the instruction sequence, and was then used to make the trigger signal. They successfully injected multiple shots using the rapidly reacting diode laser.

Lasers are used to not only inject faults but also scan and read the values of memory cells [22]. In order to read SRAM values while the chip was operating, a red 650 nm laser was used to scan the layout of the chip and draw a map along the memory address. It was found that the resistance differed when the PMOS and NMOS were closed or opened.

#### 2.2.3. Ultraviolet

**Figure 1.**
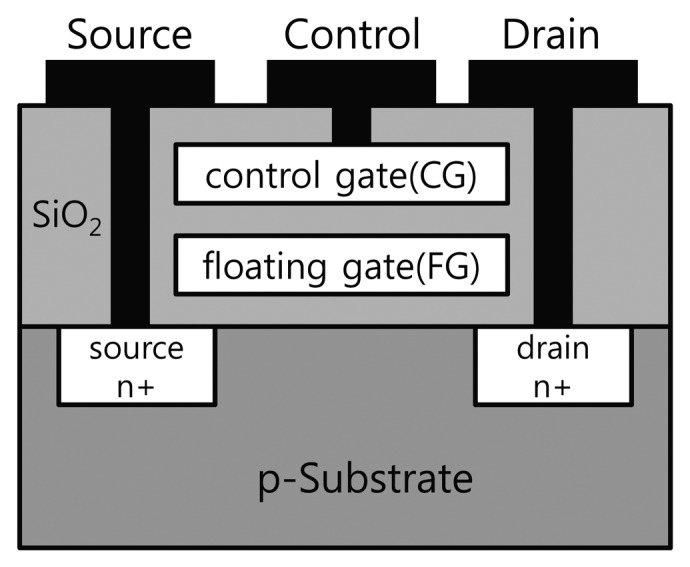
Structure of a non-volatile memory cell.

UV light has been used to alter values in a chip for many years. For instance, it is widely known that UV light was used to reset EPROM [23]. Most non-volatile memory consists of a control gate (CG), source, drain, and floating gate (FG), as shown in Figure 1. In the initial state, if a voltage is supplied to the CG, it gathers electrons between both n-diffusers, and the resulting current flows between the source and the drain. This represents the logically high state. If the electrons are locked in the FG, which is isolated by an oxide, the current flow between source and drain is disturbed by raising the threshold voltage. This represents the logically low state, which is maintained even if power is not supplied to the non-volatile memory. When the silicon is exposed to UV light, it causes photons to ionize, allowing the electrons to escape from the FG.

At first, UV light was used to reset the security fuse-bit of a microcontroller, which prevents memory from being read through the data interface [17,24]. After that, Skorobogatov [6] presented another fuse-bit attack method, applying UV-resistant ink to the top of a chip as a mask. Nowadays, most chips are resistant to these kinds of attack.

More recently, attacks have begun to target non-volatile memory. Schmidt *et al.* [25] used a 254 nm UV light to attack the CY27H010 flash memory (Cypress Semiconductors), the PIC16F54 and PIC16F84 microcontrollers (Microchip), and the AT89C2051 flash memory and ATmega48 microcontroller (ATMEL). All of these chips were decapped and their memory contents reset by UV light. The time taken to reset the memory varied from several minutes to tens of minutes. Bit flipping via UV light requires a longer time for flash memory than for SRAM. This characteristic makes a real-time attack difficult. However, it is possible to perform a precise location attack using UV-resistant markers or ink and a needle.

### 2.3. Countermeasures against Optical Fault Injection Attacks

In this subsection, we present some existing countermeasures against optical fault injection attacks.

#### 2.3.1. Metal Shielding

The most widely used method to defeat optical fault attacks is metal shielding. By covering the layout of the semiconductor with a metal plate, UV cannot reach the sensitive area. Shields can be either passive or active [8]. In the case of a passive shield, the metal layer is added after the layout has been finished. It reflects light, and prevents micro-probe from contacting the layout. Generally, passive shields can easily be removed. An active shield can sense this removal, so that once the shield becomes detached from the chip, no power is provided or the chip ceases to function. Nevertheless, this can be overcome by a precise attack, and chips are still vulnerable to optical injection from the bottom. Also, it is possible for X-ray and infrared light to penetrate a metal shield [5], but there are no reports of these sources being used for attacks.

#### 2.3.2. Dual-Rail Logic

Moore *et al.* suggested dual-rail asynchronous (self-timed) logic as a countermeasure against side-channel analysis [26]. Dual-rail logic uses 2 bits to represent a 1-bit value. In the (1-of-n) one-hot data encoding, the data transition gives the same power consumption pattern, and this property is applied in the side-channel analysis countermeasure. Dual-rail logic is a 1-of-2 one-hot encoding, *i.e*., 0 is expressed as (0,1) and 1 as (1,0), and (0,0) is used to represent the clear state. Originally, (1,1) was an unintended state due to bugs, but it was then used to represent the faulty state in the optical fault countermeasure.

One disadvantage of dual-rail logic is that it requires approximately twice the original chip area. Moreover, it is hard to validate the asynchronous circuit, and most EDA tools do not support the asynchronous circuit design.

#### 2.3.3. Light Detector (Sensor)

Light sensors, such as a photodiode, can be used to sense the intensity of light. The photodiode converts the light into a current, and this can be used to detect the opening of the IC package. However, as the photodiode is an analog device, it requires an additional cost to allow integration with a digital circuit. Also, the photodiode is larger than other devices and is easily found, making it vulnerable to a precise masking attack.

## 3. Reset Tree-Based Optical Fault Detection

In this section, we present the proposed reset tree-based optical fault detection scheme. RTOFD utilizes the buffers on a reset net, which is an essential component in most ICs, to detect an optical injection in a chip. This scheme uses the chip's built-in buffers as optical fault detection sensors, meaning that it can be implemented simply and cheaply. Also, our scheme is expected to be resistant to fault injection from the bottom of the die, which is still vulnerable when metal shielding is used to protect the top.

### 3.1. Reset Networks in Digital Logic

**Figure 2.**
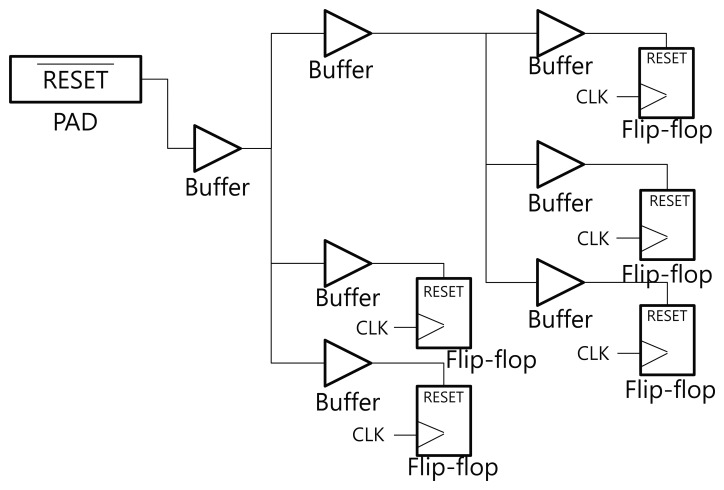
Buffer tree over the reset net.

In general, there are a number of flip-flops in a digital implementation of cryptographic hardware. All flip-flops in the circuit need to be connected with the clock and the reset signal. These signals are classified as a high fanout network (HFN). In order to propagate the reset signal to all of the scattered flip-flops, buffers are inserted into the reset net. Because the electric signals become weaker as they propagate through the metal wire, the signal needs to be recharged by the buffers. Figure 2 shows the reset signal tree in a general digital circuit. The reset signals propagate through several buffers and are sent to every reset port of the flip-flops. We propose an optical fault detection scheme that utilizes the buffers on the reset net tree. The buffer outputs can be flipped by a particle strike, and have good characteristics for use as an optical sensor.

The reset signal is usually active at the low state. Flip-flops sense the low state or falling edge of the reset signal, and place themselves into the reset state. When digital logic is operating in the normal state, the reset signal remains in the high state. If the optical fault is injected to a sensitive region of the buffer, the reset signal moves to the low state and causes the flip-flop output to be cleared. This can lead to an error, but may also be used to sense the optical fault injection.

### 3.2. Reset Tree-Based Optical Fault Detection

**Figure 3.**
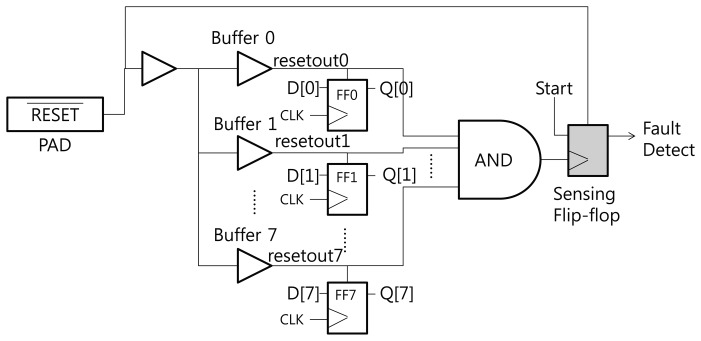
Structure of the Reset Tree-based Optical Fault Detection circuit.

Figure 3 shows the structure of the proposed RTOFD circuit. To explain our scheme, we assume that all flip-flops are reset when the reset signal has a negative edge. FF0 to FF7 denote the flip-flops that exist in the original design. All flip-flops are connected to the reset signal. Buffers are inserted to prevent signal degradation, and are denoted as Buffer 0 to 7. Normally, the buffer output is in the high state. If a laser hits a sensitive area in the buffer, the output moves to the low state. In order to sense this change, we place a sensing flip-flop in the buffers, and combine the output of several buffers using a big AND gate. The output of the AND gate is connected to the CLK port of the sensing flip-flop. This flip-flop updates its value at the negative edge of the clock signal (in this case, the combined reset signal from several buffers).

We now explain how this scheme works. Before the chip begins operation, the reset signal is turned to the low state, and all flip-flops are reset to the low state. After the reset signal switches to the high state, the start signal is triggered and the chip performs its normal operations. If an optical fault is injected and affects one of the buffers on the reset net, the output of the buffer turns to the low state. This propagates through the AND gate, triggers a negative edge in the sensing flip-flop, and finally updates the sensing flip-flop to the start (high state) signal. Through this process, we can detect optical faults.

EDA tools for synthesis, placement, and routing do not usually generate a layout like Figure 3. The EDA tools have the tendency to merge several logics or remove useless logics. In order to prevent unintended optimization from using EDA tools, appropriate design constraints should be carefully defined.

### 3.3. Simulation and Results

#### 3.3.1. Radiation-Induced Currents

Optical fault injection techniques are not a new phenomenon. Since the first semiconductors were fabricated, malfunctions caused by cosmic ray-induced currents have been observed [11], and lasers can be used to measure the currents induced by a particle strike [27]. When high-energy particles strike a sensitive region of the semiconductor, such as the p–n junction, it causes an electron-hole pair, and the resulting transient current changes the state of the circuit. The radiation-induced current is modeled as follows [11]:

(2) $$I(t)=I_{0}(e^{-t/\tau_{\alpha }}-e^{-t/\tau_{\beta }})$$

In the above equation, *I_0_* denotes the maximum charge collection current, *τ_α_* is the collection time-constant of the junction, and *τ_β_* is the ion-track establishment time constant. *I_0_* can be expressed as $\frac{Q}{\tau_{\alpha }-\tau_{\beta }}$, where *Q* is the charge deposited by the particle strike (positive or negative). *τ_α_* and *τ_β_* are the process-related factors, and *τ_α_* is usually greater than *τ_β_*.

#### 3.3.2. Simulation Method

**Figure 4.**
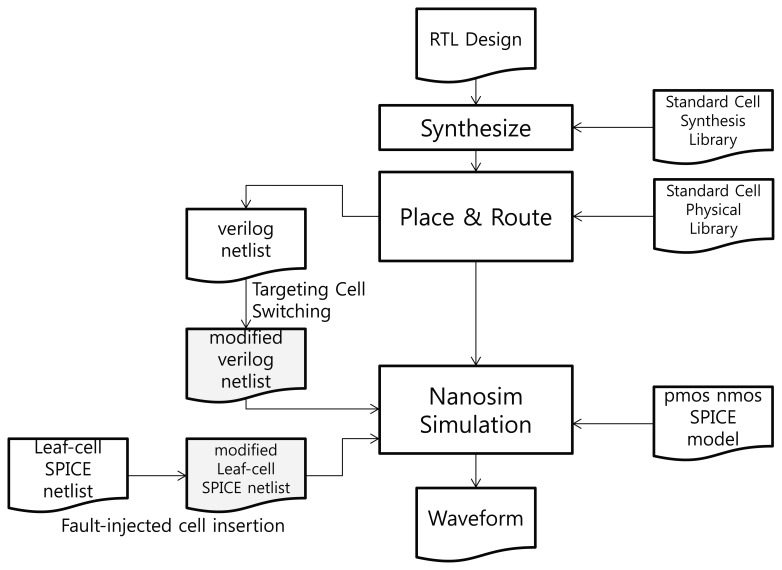
The steps and files required for the simulation.

We carried out a simulation to verify the performance of our proposed RTOFD. The steps and files required for the simulation are presented in Figure 4.

If the circuit layout is presented in a transistor-level SPICE netlist, it takes too long to simulate the circuit, and is difficult to find the location at which the fault should be injected. Thus, we used Synopsys NanoSim as a SPICE simulator. With this tool, the simulation process can be simplified by integrating the routed verilog netlist with a SPICE sub-circuit model (leaf-cell SPICE netlist) of each standard cell. To generate a verilog netlist, general chip design processes are included in the simulation steps. The Synopsys Design Compiler and IC Compiler were used for the synthesis and P&R (Place & Route). TSMC's 90 nm standard cell library was used for synthesis, P&R, and simulation. The library kit includes a synthesis and physical library, a leaf-cell SPICE netlist, and a SPICE model for PMOS and NMOS. Each standard cell's transistor-level SPICE netlist is assigned to a sub-circuit (.subckt of SPICE) in the leaf-cell netlist. NanoSim simulates the entire chip at the SPICE level by integrating the cell-level verilog netlist with the leaf-cell netlist.

**Figure 5.**
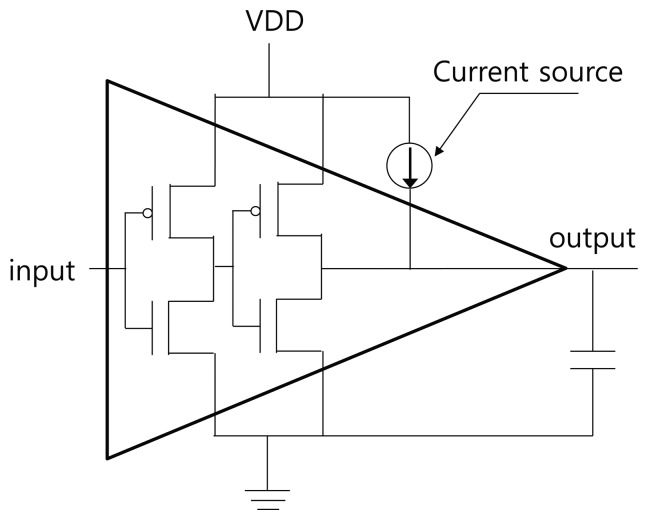
Transient current source placement.

At the register transfer level, RTOFD is synthesized, placed, and routed through the normal chip design process. After that, the cell-level verilog netlist is extracted. To inject a fault in a specific buffer, we change the cell name of the buffer by adding the suffix “F.” Next, we modify the leaf-cell SPICE netlist by duplicating the buffer sub-circuit and assigning it the same name as in the verilog netlist. A current source is then inserted to a new sub-circuit, as in Figure 5. The current source is expressed as a double exponential, as in Equation (2). The value of *τ_α_* and *τ_β_* are taken from [12]. The value of *Q* is dependent on the process technology and the type of light source. We used a value of 0.3 pC for *Q*, which is enough for the transient error. Finally, we can retrieve a waveform from the NanoSim simulation.

#### 3.3.3. Simulation Results

**Figure 6.**
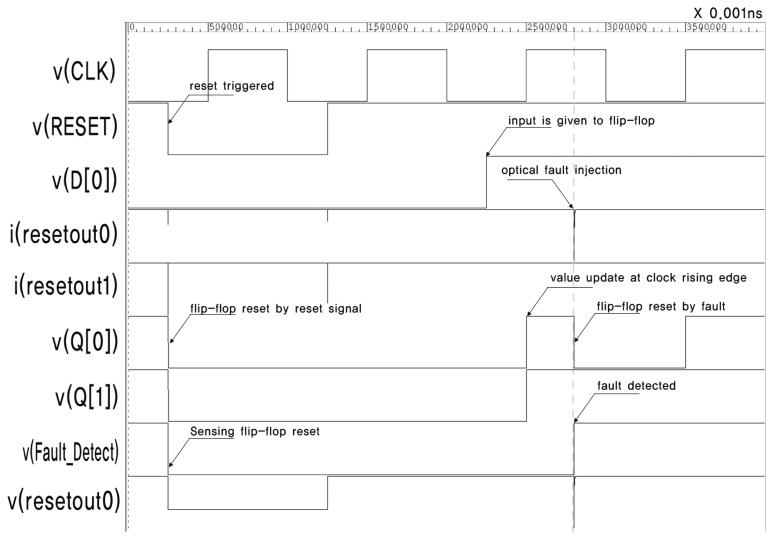
Simulation result for the circuit in Figure 3.

Figure 6 presents the simulation result of the circuit in Figure 3. *v*(*x*) and *i*(*x*) denote the voltage and current of signal *x*, respectively. There are eight flip-flops in the circuit. This is not sufficient for synthesizing an HFN, so we intentionally insert buffers over the reset net. Appropriate constraints are defined to prevent buffers from being removed by optimization. The current source, which represents the transient current due to optical fault injection, is attached to Buffer 0.

The stimuli for the simulation are as follows. The main clock is assigned a frequency of 10 MHz. The reset signal is turned off after 25 ns, and all flip-flops, including the sensing flip-flop, are reset. The reset signal is turned on again at 125 ns, and the inputs of all flip-flops, D[0]–D[7], are turned on. The outputs of all flip-flops, Q[0]–Q[7], are turned on by the positive edge of CLK. The optical fault is injected at 280 ns, and this causes a transient voltage drop in *resetout0* and the resetting of FF0. This also triggers the sensing flip-flop to be turned on. Finally, the sensing flip-flop successfully detects the injection of an optical fault. The output of the sensing flip-flop can be used to alert the fault injection or halt the operation of the chip.

**Figure 7.**
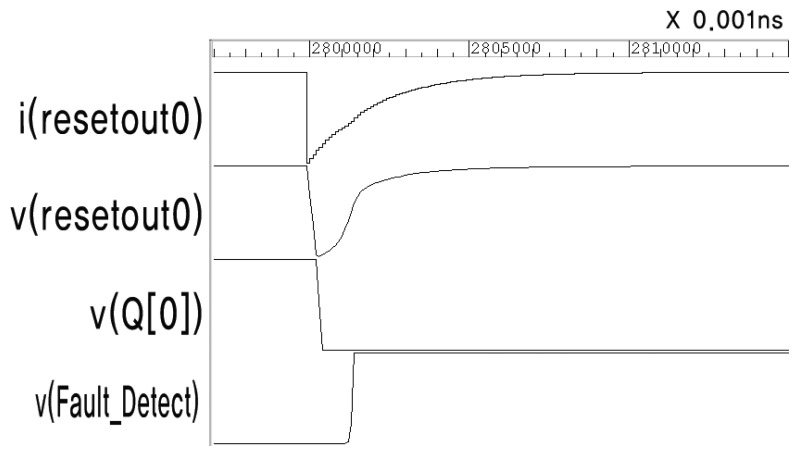
Enlarged waveform at the moment when the fault is injected.

Figure 7 shows the enlarged waveform at the moment the fault is injected. The output of *resetout0* changes according to the double exponential Equation (2). The voltage of Buffer 0 (resetout0) drops transiently due to the current change, and is then restored immediately. This causes FF0 to be reset, and the sensing flip-flop can detect this short voltage drop.

### 3.4. Alternative Structure of RTOFD

**Figure 8.**
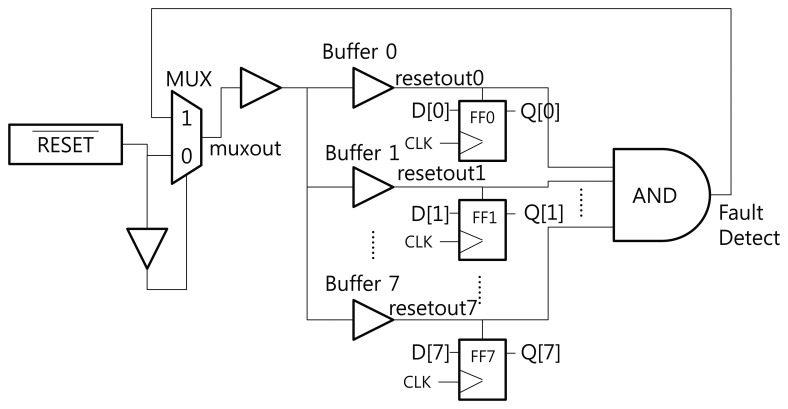
Alternative structure of RTOFD circuit.

Figure 8 shows an alternative structure for the RTOFD scheme named RTOFD-RL. This structure enables all flip-flops to be reset by positioning them in a loop. When the reset signal is turned off, all flip-flops are reset by the negative edge of the reset signal. After the signal is turned on, the outputs of each buffer also change to the high state, the output of the AND gate is chosen from MUX to form a loop, and then the buffer outputs are kept in the high state. If one of the buffer outputs is turned to low by the injected fault, this affects the whole circuit, and every flip-flop is reset by the reset loop.

**Figure 9.**
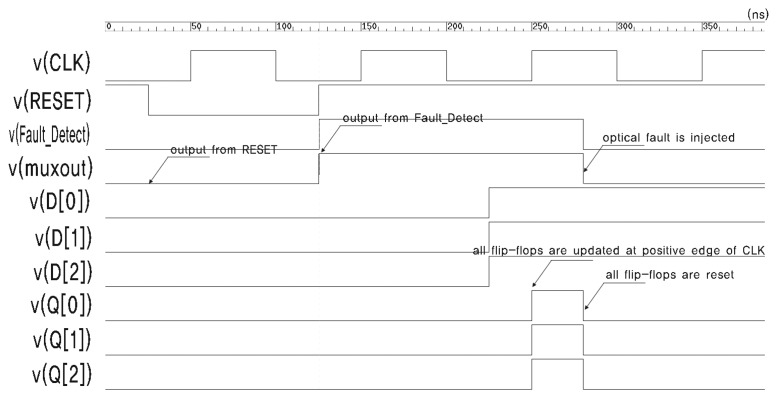
Simulation result for the circuit in Figure 8.

Figure 9 illustrates the simulation result for the alternative circuit. The stimuli are the same as the previous simulation. At 25 ns, as the reset signal changes to the low state, the reset signal itself is chosen for *muxout*. At 125 ns, as the reset signal changes to the high state, all buffers in the circuit also change to the high state, and then the output of the AND gate (Fault Detect) is chosen for *muxout* to form a loop. All flip-flops are updated to the high state at 250 ns, but are then reset at 280 ns by the optical fault injection. This scheme can be used to halt the operation of the entire chip.

## 4. Discussion

### 4.1. The Effect of Pulse Width on RTOFD

**Figure 10.**
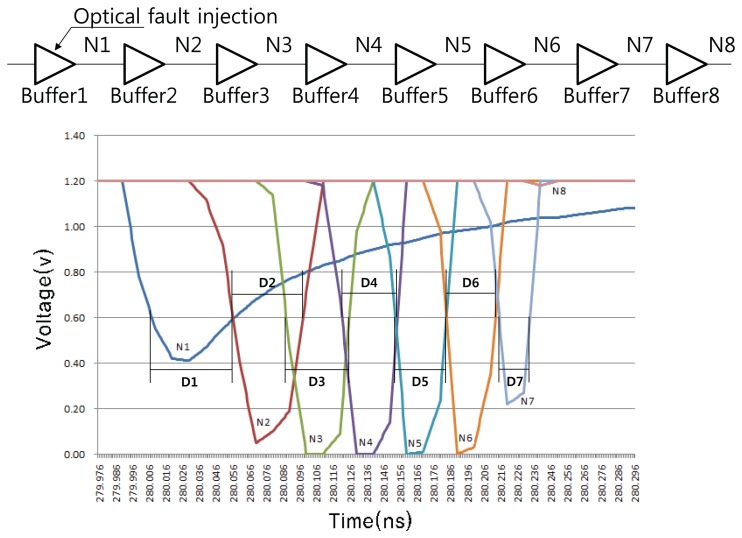
Simulation result for an optical fault injection in the buffer chain.

If the buffers form a chain, the single event transient can be degraded as it propagates through several buffers according to the propagation delay [28,29]. Figure 10 shows the degradation of pulse width through the buffer chain. In the figure, a weak optical fault (0.15 fC) is injected into Buffer 1. The time D*n* is the duration for which the output from Buffer *n* remains below *V DD*/2. It can be observed that D7 is obviously shorter than D1, and that the output from Buffer 8 does not fall below *V DD*/2. If there is a sensing flip-flop next to Buffer 8, it will not sense such a weak change.

**Figure 11.**
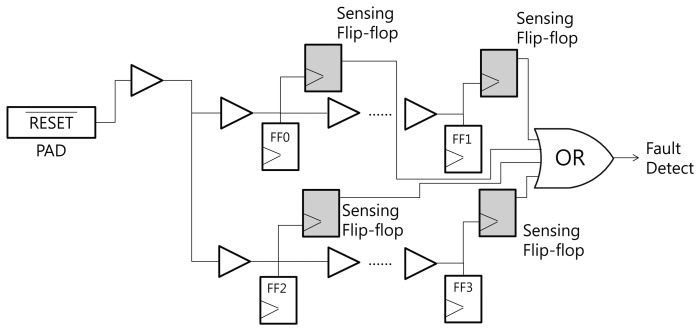
Multiple placement of sensing flip-flop.

To prevent the above situation, the sensing flip-flop can be placed at several points, as shown in Figure 11; This is called RTOFD-MS. Though it requires additional flip-flops, the average propagation path from the fault injection to the sensing flip-flop can be shortened.

### 4.2. Cost and Security Analysis

RTOFD makes use of the necessary reset signal in order to sense the transient pulse from the optical fault. Table 1 shows the cost of implementing various RTOFD schemes. We call the original scheme RTOFD, the reset loop version RTOFD-RL, and the scheme using multiple sensing flip-flops RTOFD-MS. In the table, *n* is the number of buffers connected to the big AND gate and is used to sense the optical fault injection. In general, *n* ≤ *N*, where *N* is the total number of buffers on the reset tree. RTOFD requires an *n*-to-1 AND gate, which gathers output from the *n* buffers into one signal, and a flip-flop that can sense the transition. The cost of the sensing buffers is ignored, as they are an essential component in most chips. RTOFD-RL also requires an *n*-to-1 AND gate, but a sensing flip-flop is not needed. Instead, it requires a 2-to-1 MUX and *d* buffers to produce the delay on the selected signal to MUX. Here, *d* denotes the number of buffers required to have total delays longer than the maximum delay path in the loop. RTOFD-MS requires more sensing flip-flops than RTOFD to prevent weak optical fault injection. Therefore, *n* sensing flip-flops and an *n*-to-1 AND gate are needed. Generally, the probability of successfully detecting an optical fault injection is proportional to the number of buffers *n*. That is, the more buffers used to sense the optical fault, the higher the detection probability is. If *m* additional buffers are used as sensors in order to enhance the detection probability, the extra cost of RTOFD is *m* · *S_BUFF_* + *S*_(*m* + *n*)-*to*-1*AND*_ + *S_FF_*.

Table 2 shows the cost and security vulnerabilities of existing countermeasures. Metal shielding requires an additional metal layer, but is still susceptible to attacks from the bottom of the chip. In contrast, the RTOFD scheme is expected to be effective against bottom side attacks, because the strength of the light causing a transient error will have the same effect on the buffer cells. The metal shield could also be removed, and may allow some of the light beam to penetrate [6]. The dual-rail logic method requires approximately twice the original circuit area, whereas RTOFD can be implemented at a relatively small area cost. The cost of the photodiode method can be defined as the sum of the photodiode area, which is denoted by *p · S_photodiode_*, and the logic needed to combine several signals from the photodiodes, *S_COMB_*. As RTOFD uses built-in buffers and only a little additional logic, it has a lower cost than the photodiode method. Considering its vulnerability to a simple masking attack and the difficulty of integrating analog photodiodes, the photodiode method is neither as efficient nor as secure as the proposed RTOFD scheme.

### 4.3. Limitations of the RTOFD Scheme

Though our proposed scheme is efficient in chip cost and more secure than other countermeasures, it also has a disadvantage. Our scheme can be applied only to a digital circuit that uses a reset signal. Therefore, our scheme cannot be applied to a circuit like a memory cell. For this reason, our scheme should not be used alone, but could be used to improve safety from optical fault injection attacks. Hence, the proposed scheme can be used together with other countermeasures, such as metal shielding, dual-rail logic, and photodiodes.

## 5. Conclusions

In this paper, we proposed an optical fault detection scheme that utilizes the buffers in the reset net as an optical fault sensor. As the reset net and the resulting buffers are essential components of ICs, the RTOFD scheme can be implemented with a small area overhead. The proposed scheme was implemented with EDA tools and verified through a series of simulations.

The effect of RTOFD may be degraded if a weak optical fault is injected, because the transient pulse decreases as it passes through the buffers. To overcome this, we presented a variant of RTOFD using multiple sensing flip-flops. This shortened the average path from the buffers to the sensing flip-flop.

RTOFD is more efficient and secure than existing countermeasures such as metal shielding, dual-rail logic, and photodiodes. Unlike metal shielding schemes, RTOFD can detect a light injection from the bottom of the silicon device. Also, it is difficult to mask the buffers in the reset net because they are scattered across the chip. In contrast to dual-rail logic, which requires approximately twice the original chip area, RTOFD requires only a small additional area for the logic of the scheme. Since it cannot be applied to circuits without reset signal, it could be more helpful to use it with other countermeasures.

## Figures and Tables

**Table 1.** Cost comparison of several versions of RTOFD. *S_COMP_* denotes the area cost of a specific component *COMP*.

**Scheme**	**Cost**
RTOFD	*S_n–to–_*_1_*_AND_* + *S_FF_*
RTOFD-RL	*S_n–to_*_–1_*_AND_* + *S*_2–_*_to_*_–1_*_MUX_* + *d* · *S_BUFF_*
RTOFD-MS	*S_n–to–_*_1_*_OR_* + *n* · *S_FF_*

**Table 2.** Cost and security vulnerabilities of existing countermeasures. *S_COMB_* denotes the area cost of logics for combining the photodiode signals.

**Scheme**	**Cost**	**Security vulnerabilities**
Metal Shield	additional metal layer	- susceptible to bottom side attacks - shield can be removed
Dual Rail Logic	2× chip size	
Photodiode	*p·S_photodiode_* + *S_COMB_*	- photodiode is easily found and masked
